# A Three-Arm Randomised Controlled Trial of High- and Low-Intensity Implementation Strategies to Support Centre-Based Childcare Service Implementation of Nutrition Guidelines: 12-Month Follow-Up

**DOI:** 10.3390/ijerph17134664

**Published:** 2020-06-29

**Authors:** Alice Grady, Kirsty Seward, Meghan Finch, Luke Wolfenden, Rebecca Wyse, John Wiggers, Christophe Lecathelinais, Sze Lin Yoong

**Affiliations:** 1 School of Medicine and Public Health, University of Newcastle, Callaghan 2308, Australia; kirsty.seward@health.nsw.gov.au (K.S.); meghan.finch@health.nsw.gov.au (M.F.); luke.wolfenden@health.nsw.gov.au (L.W.); rebecca.wyse@health.nsw.gov.au (R.W.); john.wiggers@health.nsw.gov.au (J.W.); serene.yoong@health.nsw.gov.au (S.L.Y.); 2 Hunter New England Local Health District, Population Health, Wallsend 2287, Australia; Christophe.Lecathelinais@health.nsw.gov.au; 3 Hunter Medical Research Institute, New Lambton Heights, Newcastle 2305, Australia; 4 Priority Research Centre for Health Behaviour, University of Newcastle, Callaghan 2308, Australia

**Keywords:** childcare, nutrition, children, diet, implementation, guidelines, menu

## Abstract

The study aimed to compare the effectiveness of a suite of implementation strategies of varying intensities on centre-based childcare service implementation of nutrition guideline recommendations at 12-month follow-up. A six-month three-arm parallel group randomised controlled trial was undertaken with 69 services, randomised to one of three arms: high-intensity strategies (executive support; group face-to-face training; provision of resources; multiple rounds of audit and feedback; ongoing face-to-face and phone support); low-intensity strategies (group face-to-face training; provision of resources; single round of audit and feedback); or usual care control. Across all study arms, only three high-intensity services were compliant with overall nutrition guidelines. A significant group interaction was found between the three arms for compliance with individual food groups. Relative to control, a significantly greater proportion of low-intensity services were compliant with dairy, and a significantly greater proportion of high-intensity services were compliant with fruit, vegetables, dairy, breads and cereals, and discretionary foods. No significant differences between the high- and low-intensity for individual food group compliance were found. High-intensity implementation strategies may be effective in supporting childcare service implementation of individual food group recommendations. Further research is warranted to identify strategies effective in increasing overall nutrition compliance.

## 1. Introduction

In 2017, the Global Burden of Disease study reported that more than 11 million deaths and 255 million disability-adjusted life years worldwide were due to dietary risk factors alone, including low fruit and wholegrain food intake, and high sodium intake [1]. Evidence shows that dietary patterns and food preferences established in childhood track into adulthood, increasing the risk of future chronic disease [2]. As such, the World Health Organization encourages countries to develop national nutrition policies and dietary guidelines with recommendations for healthy eating behaviours in childhood, as a disease prevention strategy at a population level [3,4].

Early childhood education and care services (hereby referred to as childcare services) provide access to a large number of children aged 0–6 years, at a critical stage of dietary habit development [5]. In Australia, 767,400 children aged 0–5 years attend some type of formal childcare [6] where they can consume up to 67% of their recommended daily dietary intake [7]. Evidence suggests that children’s dietary intake can be improved by interventions targeting the nutrition environments of childcare services [8,9]. Childcare services are subject to menu dietary guidelines [10,11] regarding the quantities and types of food and drinks provided to children and, thus, provide an opportunistic setting for public health nutrition interventions to improve guideline implementation.

Despite the existence of setting-specific dietary guidelines, previous national and international research has identified that such recommendations are poorly implemented. For example, a 2017 study assessing 57 childcare service menus in New Zealand reported only 5% of menus met criteria relating to quality, variety and quality of foods [12]. In Australia, a 2017 audit of 70 menus from childcare services across New South Wales (NSW) determined that none of the menus were compliant with nutrition guidelines [13]. Furthermore, none of the menus provided adequate servings of vegetables or meat/meat alternatives, and 76% provided discretionary foods (i.e., foods high in kilojoules, saturated fat, added sugars and added salt) [13].

Childcare service staff report substantial barriers to achieving compliance with recommendations, most commonly around skills, knowledge, resources and managerial support [13,14,15,16]. Such challenges to implementation need to be addressed in order for dietary recommendations to produce improvements in child nutritional intake. Despite this, a 2020 Cochrane systematic review [17] identified only two randomised controlled trials (RCTs) reporting findings of interventions to improve the implementation of dietary guidelines regarding food and drink provision to children by childcare services [18,19]. One further RCT has been identified reporting the effect of a childcare-based intervention on menu compliance with dietary guidelines [20]. The first, a US intervention which included nutrition curriculum; professional development for staff; food service recommendations; and family, healthcare provider, and community grocery store engagement, reported no significant improvements in food provision at two year follow-up [19]. 

One of the remaining RCTs is an Australian trial by Finch and colleagues, which tested the effects of a low-intensity implementation strategy, including the provision of staff training and resources and a single round of audit and feedback, and found no improvements in overall menu or individual food group compliance among the intervention arm compared to control at six-month follow-up [20]. The final trial by Seward and colleagues, also undertaken in Australia, tested the effects of a higher-intensity implementation strategy, designed to overcome barriers to the implementation of nutrition guidelines [18]. The intervention included the provision of staff training and resources, multiple rounds of audit and feedback, securing executive support, and ongoing face-to-face and telephone support. While there were no differences between intervention and control groups in overall menu compliance with guidelines, the study reported significant improvements in the intervention arm for compliance with five out of six individual food groups at six-month follow-up, suggesting that the intervention had improved food provision to some extent. 

To date, short-term (six-month) changes in menu composition have been reported for the two Australian trials [18,20], however, the sustainability of these effects are unknown. As such, longer periods of follow-up are required to assess whether these effects attenuate over time. Whilst the outcomes from the two trials were reported separately as two-arm RCTs, the trials were conducted concurrently, service randomisation occurred in parallel, and the studies shared a control arm. This provides an opportunity for comparison between the three groups within the trials. Given that the interventions in each trial varied in intensity and strategies, measured the same primary outcomes and had the same follow-up periods, comparison of the interventions as a three-arm RCT would be particularly useful to help inform policy and health practitioner decisions regarding the intensity of implementation support, and hence public health practice and resource allocation. As such, this study aimed to compare the effectiveness of a suite of implementation strategies of varying intensities (high and low) on childcare service (i) overall; and (ii) individual food group, compliance with nutrition guidelines at 12-month follow-up. The impact of the intervention on the mean number of food groups compliant with guidelines, and the mean servings of individual food groups was also assessed.

## 2. Materials and Methods 

### 2.1. Ethical Approval

Ethical approval was obtained from the Hunter New England (06/07/26/4.04) and the University of Newcastle (H-2012-0321) Human Research Ethics Committees. The component trials were prospectively registered with the Australian New Zealand Clinical Trials Registry (ACTRN12615001032549 and ACTRN12615001058561). The study is reported according to the Consolidated Standards of Reporting Trials (CONSORT) Statement and the Template for Intervention Description and Replication (TIDieR) [21].

### 2.2. Design and Setting

A three-arm RCT was conducted with centre-based childcare services within the Hunter New England Local Health District of NSW, Australia, that prepare and provide all food and drink to children while in care. The Hunter New England Local Health District encompases 368 childcare services, of which 106 are long day care services that prepare and provide food to children onsite. This region is also the location of one of Australia’s only ‘implementation laboratories’ in which the research team are embedded within the local health service [22]. In Australia, centre-based childcare refers to any licensed facility that provides care to children aged zero to six years outside the home [23]. The protocol of the high-intensity trial [24] and the six-month menu compliance outcomes for both the high and low-intensity trials [18,20] have been published separately elsewhere, including data regarding delivery of the intervention strategies (intervention uptake and fidelity). This paper reports the 12-month outcomes from participants in the three study arms, in their original allocations. 

#### 2.2.1. Participants

To be eligible, childcare services were required to prepare and provide at least one main meal (lunch) and two mid-meals (morning tea and afternoon tea) to children while in care, and be open for a minimum of eight hours per day. Services were ineligible if they did not prepare and provide meals to children, or did not have a cook with responsibility for planning the service menu (i.e., externally catered services). Those services catering exclusively for children requiring specialist care, and mobile preschools and family day care services were excluded, given the different operational characteristics of these services compared to centre-based childcare services.

#### 2.2.2. Recruitment Procedures

Recruitment for all three arms of the trial took place from October to December 2015. All potentially eligible childcare services within the region were mailed a study package (information statement and consent form) approximately one week prior to receiving a call from a research assistant to confirm eligibility and obtain consent. All childcare services were called in a random order determined via a random number function in Microsoft Excel. 

#### 2.2.3. Randomisation and Allocation

Immediately following the provision of consent via phone, using a centrally concealed random allocation procedure (i.e., concealed enveloped), childcare services were randomly allocated to one of the three study arms (high-intensity intervention, low-intensity intervention or control group) by the research team. Services were randomised in a 1:1:1 ratio via block randomisation (block sizes ranged from 2–6) using a random number function in SAS statistical software (version 9.3). Due to the nature of the intervention, childcare services were aware of their group allocation, however all trial outcome data collectors and assessors were blinded to allocation.

### 2.3. Interventions

#### 2.3.1. Intervention Development

The six-month implementation strategies for the high- and low-intensity interventions aimed to increase the implementation of recommendations outlined in the Caring for Children resource [25]. The Caring for Children resource outlines the menu dietary guidelines regarding the quantities and types of foods to be provided to children, developed specifically for childcare services within NSW, Australia. Both the high- and low-intensity interventions were delivered to all staff within participating childcare services given the supporting roles educators and other staff play in the planning of menus [13]. In particular, the interventions targeted service managers, given their operational leadership role, and cooks, given their primary role in menu planning and food preparation within these services. Both interventions were developed by an experienced team of implementation scientists, behavioural scientists, health promotion practitioners, and dietitians, in consultation with childcare service cooks and service managers [22]. The interventions were designed to address the identified barriers and enablers to the implementation of the menu dietary guidelines based on the Theoretical Domains Framework [26] (TDF) and previous research conducted in the childcare setting [14,17,27,28].

The TDF is a theoretical framework of determinants considered to influence behaviour, which may enable or impede the implementation of evidence-based practice [29,30,31,32]. The TDF incorporates 33 theoretical models and frameworks of behaviour change, and has been empirically validated in the childcare setting, as well as healthcare settings [26,28,29]. The TDF was applied in this study to provide a systematic and theoretical guide in which to both assess the barriers and enablers to implementing nutrition guidelines in the setting, and to identify the potential behavioural change factors to be targeted in order to improve guideline implementation. As part of the intervention strategy development, a semi-structured interview based on the TDF was conducted with staff of seven childcare services, to identify relevant settings-based barriers and enablers to implementation of menu dietary guidelines. Such an approach was chosen as qualitative research can provide additional information to assist in understanding processes, context and complexity, that quantitative approaches alone may not [33]. Selection and design of both the high- and low-intensity implementation interventions was informed by the findings of these semi-structured interviews, systematic review evidence [13,34], and onsite observations of menu-planning practices. This resulted in identification of a number of determinants of guideline implementation, namely limited knowledge of servings required, limited skills in planning compliant menus and the ability to self-assess compliance, limited experience planning for implementation, limited managerial support, and limited resources [18,20]. The content and strategies of each intervention were adapted for different intervention intensities. While both interventions were designed to improve service menu compliance with dietary guidelines, the selection of implementation strategies identified in the low-intensity intervention sought also to consider the costs and pragmatics of implementation from the health service perspective. Considerations resulted in the targeting of a smaller range of barriers, and using less resource-intensive implementation strategies. Further details regarding the process for selection of implementation strategies are reported elsewhere [18,20,24].

#### 2.3.2. Implementation Strategies

Table 1 provides details of the implementation strategies for the two interventions. The Seward intervention [18] (higher-intensity) contained additional strategies and a higher strategy dose to that of Finch [20] (lower-intensity). To ensure consistent language, interpretation and comparability of implementation strategies to the wider implementation research literature, the implementation strategies employed within the current study are described using the Expert Recommendations for Implementing Change (ERIC) taxonomy [35]. Detailed application of these strategies are described using the Proctor framework [36] to facilitate replication. All strategies were provided within the first six months of the study period. Implementation support officers responsible for delivering the intervention were all trained health promotion staff whom had at least five years’ experience working as a dietitian or in obesity-prevention initiatives in the early childhood education and care setting. Furthermore, detailed descriptions are reported elsewhere [18,20,24].

#### 2.3.3. Control Group

Services randomised to the control group were mailed a hard copy of the Caring for Children resource [25] within the first month of randomisation and were provided with usual care from the local health district. Usual care included general telephone support from a health promotion officer upon request to implement the NSW state-wide obesity prevention program [45]. Control services did not receive any further implementation support during the intervention period.

### 2.4. Measures 

Baseline and follow-up data collection was conducted concurrently for all three arms of the trial. Baseline data collection took place between November 2015 and February 2016 with follow-up data collection approximately 12 months later (from February to May 2017). While the component trials were prospectively registered, the 12-month outcomes for the low-intensity intervention were not due to resource constraints at the time of registration. Outcomes across all three study arms were assessed using the same method described below. 

#### 2.4.1. Primary Outcomes: Compliance with Nutrition Guidelines

Menu compliance with nutrition guidelines was assessed via detailed menu assessment undertaken by a trained dietitian in accordance with best practice protocols at baseline and follow-up [24,46]. A detailed description of the comprehensive menu review process is described in the protocol paper [24]. Nutrition guidelines for the sector [25] recommend menus provide at least 50% of the recommended daily servings of the five food groups specified in the Australian Guide to Healthy Eating (AGHE) [10] across a two-week menu cycle (10 days). Specifically, it is recommended that service menus provide the following servings per child every day within a two-week period, at a minimum: (i) vegetables and legumes/beans (two servings); (ii) fruit (one serving); (iii) wholegrain cereal foods and breads (two servings); (iv) lean meat and poultry, fish, eggs, tofu, seeds and legumes (three-quarter servings); and (v) milk, yoghurt, cheese and alternatives (one serving) [25]. Compliance with nutrition guidelines was determined based on the calculations of servings of each food group provided per child each day. The calculated servings of each food group were rounded to the nearest 0.25 of a serving based on dietitian consensus of a meaningful portion of a serving at a population level. Rounding did not change the overall nutrition guidelines or food group compliance outcomes.

Primary trial outcomes were assessed:

1. Overall compliance with nutrition guidelines. Overall menu compliance was defined as the proportion of services providing the minimum recommended number of servings according to the nutrition guidelines for all five AGHE food groups for every child, every day over a two-week menu (10 days).
2. Compliance with nutrition guidelines for individual food groups. Individual food group compliance was defined as the proportion of services providing the minimum recommended number of servings compliant with the nutrition guidelines for each of the five individual AGHE food groups, plus discretionary foods, for every child, every day over a two-week menu (10 days). The nutrition guidelines for the sector recommend childcare services do not provide discretionary foods. In order to be deemed compliant a service needed to have zero discretionary foods on the menu (i.e., if a service provided any discretionary foods on the two-week menu, they were not deemed as compliant).

Two secondary outcomes were included to provide greater description of any changes occurring to the menu. These measures were not prospectively registered:

3. Menu compliance score (mean number of individual food groups compliant). A score for menu compliance was calculated by summing the number of food groups and discretionary foods provided in sufficient quantity to meet guideline recommendations for each service. Scores could range between 0 and 6, with a score of 1 allocated for each of the five AGHE food groups and discretionary foods that were compliant (e.g., if a service provided adequate servings for all food groups, including zero discretionary foods, they scored a 6).
4. Mean number of servings of each individual food group provided. The mean number of servings of each AGHE food group and discretionary foods provided on the menu was assessed.

#### 2.4.2. Childcare Service Operational Characteristics 

At baseline, service managers were asked to complete a mailed pen and paper survey which collected service operational data including the number of children the service provides food for each day and service postcode (to determine service socioeconomic status). Survey items have been used in previous Australian surveys of childcare service managers conducted by the research team [47,48]. This data was collected in order to describe the study sample, and to compare service characteristics between study arms.

#### 2.4.3. Service Cook Demographics 

At baseline, service cooks were asked to complete a mailed pen and paper survey which collected demographic data including education level, years employed as a cook in the childcare setting, age and weekly hours worked. Survey items were adapted from a previous state-based survey of childcare services conducted by the research team [47]. 

#### 2.4.4. Sample Size and Power Calculations

Separate a priori sample size calculations for both the high- and low-intensity interventions determined a sample of 29 services in the intervention and 29 services in the control would enable detection of an absolute difference of 32% between groups in the proportion of services with overall nutrition guideline compliance at follow-up, with 80% power, with a two-sided alpha of 0.05, assuming a prevalence of 13% in the control arm. A post-hoc minimum detectible difference calculation determined that this sample size would enable detection of an absolute difference of 34% between the high- and low-intensity interventions in the proportion of services with overall compliance at follow-up, with 80% power, with a two-sided alpha of 0.05, assuming 50% prevalence in the low-intensity arm.

### 2.5. Statistical Analyses 

SAS (version 9.3, SAS Institute, Cary, North Carolina, USA) software was utilised. All statistical analyses were undertaken by a statistician blinded to group allocation. All statistical tests were 2-tailed with an alpha value of 0.05. All outcomes were analysed under an intention-to-treat framework using all available data with services analysed based on the groups to which they were allocated, regardless of the treatment type or exposure received. For each primary and secondary outcome, an exact logistic regression model and linear regression model (respectively), adjusted for the baseline value of the outcome, was used to determine whether there was an association between group allocation and food group compliance at 12 month follow up, as well as examine the effectiveness of each intervention, relative to the control and to each other, in improving compliance with nutrition guidelines for individual AGHE food groups. Analyses using multiple imputations for missing data were also performed using the MI procedure in SAS. 

Descriptive statistics were used to describe the service operational and cooks’ characteristics. Service postcodes classified as being in the bottom 50% of NSW according to the Socio-Economic Indices for Areas [49], were classified as lower socioeconomic status. Service geographic locality was classified as either major city and inner regional or outer regional and remote according to the Australian Statistical Geography Standard [50]. Analyses of variance (ANOVAs) were used to compare service and cook characteristics between intervention and control groups at baseline, and the characteristics associated with completion of the 12-month follow-up data collection (i.e., those not lost to follow-up).

## 3. Results

**Figure 1:**
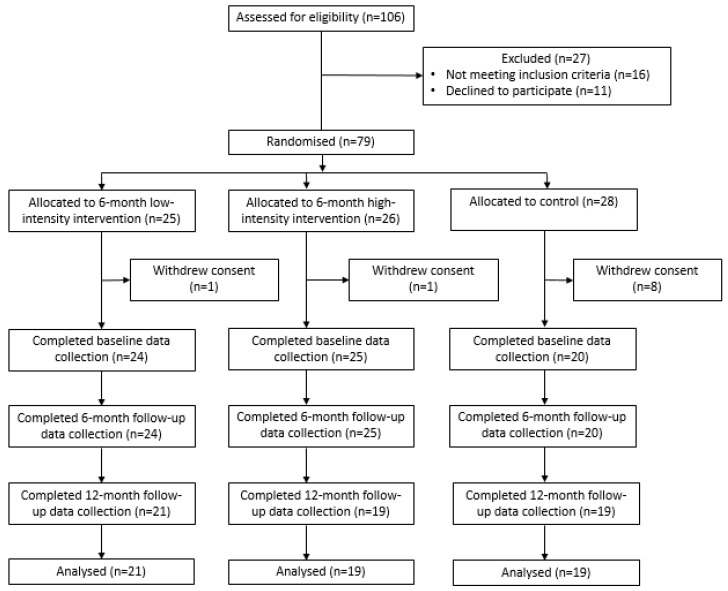
Study Consolidated Standards of Reporting Trials (CONSORT) flow diagram.

Of the 106 potentially eligible childcare services in the study region, 90 (85%) were eligible, 79 (87%) provided consent to participate in the study and were randomised (high-intensity intervention *n* = 26; low-intensity intervention *n* = 25, control *n* = 28) (Figure 1). Of these services, 10 (low-intensity = 1; high-intensity = 1; control = 8) withdrew consent prior to baseline data collection, without knowledge of group allocation. Reasons for withdrawal for each service were not systematically recorded. Of the remaining 69 services, 10 did not consent to 12-month follow-up data collection (low-intensity = 3; high-intensity = 6; control *n* = 1). Reasons for non-consent for each service were not systematically recorded. Baseline characteristics of participating childcare services and service cooks are described in Table 2. Services in the control arm had a significantly higher proportion of cooks with a university or technical and further education (TAFE) qualification compared to services in either intervention (*p* = 0.02). There were no service or cook characteristics associated with completion of 12-month follow-up data collection.

### 3.1. Primary Outcomes at 12 Months

#### 3.1.1. Overall Compliance with Nutrition Guidelines

At 12-month follow-up, three high-intensity intervention services (16%), zero low-intensity intervention services, and zero control services were fully compliant with the nutrition guidelines for the sector (Table 3). Statistical analyses were not performed given zero values across multiple cells. 

#### 3.1.2. Compliance with Nutrition Guidelines for Individual Food Groups

- *Group interaction:* significant differences in the proportion of services compliant with individual food groups were found between groups for vegetables; fruit; breads and cereals; dairy; and discretionary food (Table 3). Following multiple imputation, the difference in compliance for breads and cereals was no longer statistically significant.
- *Low-intensity* vs. *control:* relative to control, a significantly greater proportion of services allocated to the low-intensity intervention were compliant for the dairy food group (Table 3). Multiple imputation did not result in any changes to statistical significance for these analyses.
- *High-intensity* vs. *control:* relative to control, a significantly greater proportion of services allocated to the high-intensity intervention were compliant for five of the six food groups (vegetables; fruit; breads and cereals; dairy; and discretionary food) (Table 3). Following multiple imputations, the difference in compliance for breads and cereals was no longer statistically significant (odds ratio (OR) = 6.98; 95% confidence interval (CI): 0.72, 67.24; *p* = 0.09).
- *Low-intensity* vs. *high-intensity:* pairwise comparisons indicated there was no significant difference between the high- and low-intensity interventions for compliance with any food group (Table 3). Multiple imputation did not result in any changes to statistical significance for these analyses.

#### 3.1.3. Menu Compliance Score (Mean Number of Individual Food Groups Compliant)

- *Group interaction:* a significant difference in the mean number of food groups compliant with guidelines was found between groups (Table 4).
- *Low-intensity* vs. *control:* relative to control, a significantly greater number of food groups were compliant with guidelines in the low-intensity intervention services (Table 4).
- *High-intensity* vs. *control:* relative to control, a significantly greater number of food groups were compliant with guidelines in the high-intensity intervention services (Table 4).

*Low-intensity* vs. *high-intensity:* pairwise comparisons indicated there was no significant difference between the high- and low-intensity interventions for the mean number of food groups compliant on the menu (Table 4).
Multiple imputation did not result in any changes to statistical significance for these analyses.

#### 3.1.4. Servings of Individual Food Groups

- Group interaction: significant differences in the servings of individual food groups were found between groups for vegetables; fruit; dairy; and discretionary (Table 4).
- Low-intensity vs. control: relative to control, a significant increase in servings of fruit; dairy; and discretionary was found in the low-intensity intervention (Table 4).
- High-intensity vs. control: relative to control, a significant increase in servings of four out of six food groups (vegetables; fruit; dairy; and discretionary) was found in the high-intensity intervention (Table 4).
- Low-intensity vs. high-intensity: pairwise comparisons indicated there was a significant difference between the high- and low-intensity interventions for servings of vegetables (Table 4).

Multiple imputation did not result in any changes to statistical significance for these analyses.

## 4. Discussion

This study is the first three-arm RCT to compare the effectiveness of high- and low-intensity implementation strategies in improving childcare service compliance with nutrition guidelines for the sector. The study found that neither intervention resulted in a measureable difference in overall nutrition guideline compliance. However, the study also found significant differences in compliance with nutrition guidelines between groups for five out of six individual food groups. These findings provide guidance to policy and practice decision makers responsible for the implementation and sustainability of obesity prevention initiatives in the childcare setting.

At 12-month follow-up the study found only three services in the high-intensity intervention to be compliant with overall nutrition guidelines, and none in the low-intensity and control arms. These findings are similar to those reported at the six-month follow-up [18,20], and findings from controlled trials included in a Cochrane systematic review [17], suggesting that the delivery of highly intensive support to childcare services produces compliance in only a small proportion (16%) of services. As such, it is likely that for the majority of services, additional and/or alternate implementation strategies and support are required for a longer duration, in order to increase overall compliance among a greater number of services. 

The difficulty in achieving full compliance with guidelines, even with intensive implementation support also suggests that the nutrition guidelines for the sector may be overly complex, and represent an unrealistic goal for services to achieve [15,51,52], particularly when there are no requirements for service cooks to have nutrition qualifications. Given such challenges, review of the feasibility and fit of current sector nutrition guidelines with childcare service accreditation requirements is warranted. Statistically significant improvements in the mean number of foods groups compliant with guidelines were found for both the high- and low-intensity interventions, relative to control. As such, improvements to the food provided to children in care may be more likely to be achieved and sustained when modest goals to improve compliance are set [53], rather than an emphasis on overall compliance with nutritional guidelines. In line with this, an incremental approach to implementation may reduce demoralization on the part of childcare services unable to achieve full compliance despite significant investment and effort. Alternatively, it is possible that the selection of implementation strategies do not adequately target the complex calculations required to plan a menu that have been previously reported as significant barriers by childcare staff [13,52,54]. Online approaches that allow services to self-assess and provide suggestions to modify menus, such as those currently being tested by the research team [55,56], could provide an alternate strategy to address such challenges in achieving full compliance.

This study found significant differences between arms in terms of compliance with nutrition guidelines for, and servings of, individual food groups. Specifically, a significant difference in the proportion of the low-intensity and the high-intensity intervention services compliant with recommendations for dairy, compared to control, was observed. While there were only slight increases in compliance and mean servings in the low-intensity group for this food group, a decrease in food group compliance and mean servings was observed in the control group. The mean servings of dairy provided per child, per day were above the recommendations (one serving). This suggests a lack of consistency in the amounts provided across each day of the menu, and it is likely that services were compliant on some, but not all days of the two-week menu (as required for compliance). Significant differences in servings of fruit and discretionary foods were also observed between the low-intensity intervention and control. The high-intensity intervention significantly increased compliance among five of the six food groups (vegetables; fruit; breads and cereals; dairy; and discretionary foods), compared to control; with significant differences in servings for four of these food groups (vegetables; fruit; dairy; and discretionary) also observed. Interestingly, there were no significant differences in food group compliance observed between the low-intensity and the high-intensity intervention, with a significant difference observed only for the servings of vegetables in the high-intensity intervention. Neither intervention produced a statistically significant improvement in compliance or servings for the meat/meat alternatives food group. This finding may suggest there are additional barriers to implementation for this particular food group, which could include costs, dietary restrictions (e.g., vegetarian and vegan diets) or allergies (e.g., eggs) that limit menu options. Collectively, the variable impact of the high- and low-intensity interventions on individual food groups suggests that alternate and tailored strategies for each food group may be needed. Future research should investigate this hypothesis. 

These findings indicate that while some improvements in individual food group compliance can be achieved by low-intensity support, high intensity-interventions are required to produce substantial improvements in a greater number of food groups. Such findings are similar to menu interventions of varying intensity conducted in the school canteen setting, which found the greater the intensity of the implementation strategy, the greater the effect size on canteen menu compliance with policies [56,57,58,59]. Furthermore, the finding that four out of six individual food groups significantly improved in both compliance and servings in the high-intensity arm, compared to control, is in line with a previous review which suggests an upper limit of 80% implementation of public health programs is common [60,61]. 

When comparing the current results to those reported immediately post-intervention (six- month follow-up) within the high-intensity intervention [18], a significant improvement remained stable for the fruit, dairy and discretionary food groups at the 12-month follow-up compared to control, indicating that the outcomes achieved directly after the end of the intervention were able to be sustained by childcare services in the absence of any further implementation support. In addition to this, the proportion of services compliant with the vegetables and breads and cereals food groups increased, also becoming statistically significant at 12-month follow-up. The intervention effect for the meat/meat alternatives food group, however, was no longer significant at 12-month follow-up. For the six-month outcomes of low-intensity intervention [20], improvements in compliance for the dairy food group became statistically significant at 12-month follow-up, compared to control. Food group compliance in the control group decreased from six to 12 months for fruit, breads and cereals, meat/meat alternatives and dairy. This suggests the significant findings for some of the food groups are the result of both the control arm reducing compliance and the intervention arms increasing. In light of this, the interventions may be viewed as preventative of a decline in compliance, further warranting the need for sustained support for childcare services to implement the nutrition guidelines. 

Findings from non-controlled and non-randomised trials within the childcare setting report similar findings, more broadly. That is, there is inconsistent evidence of the effectiveness of implementation strategies in improving the implementation of nutrition policies, practices and programs in childcare services [17]. Given such findings, the collection and reporting of contextual information, for example intervention acceptability, delivery and costs, is recommended to allow for a deeper interpretation of outcomes [62]. While intervention acceptability and delivery of the implementation strategies at six months were reported as high for both the low- and high-intensity interventions [18,20], additional contextual measures at 12-month follow-up, such as acceptability of the intervention dose and timeframe of support, and long-term sustainability of the intervention from the perspectives of service managers and cooks, may have provided further insights into the current findings. Contamination was also examined and reported immediately post-intervention at six months follow-up [18]. While no services reported receiving any additional intervention or support beyond the prescribed intervention at that time, internal records continually maintained by the research team identified one control service received one round of written feedback on their menu between six to 12 months, which may have had an impact on study findings. Given the investment in designing interventions that directly address identified barriers and enablers to the implementation of menu dietary guidelines according to the TDF, future research examining barriers and enablers as potential mediators of both short-term and long-term changes in menu and food group compliance outcomes is also suggested. This would enable an assessment of the mechanisms of change in which the selected implementation strategies exert their effort, and whether these mechanisms are constant or changing over time [63]. Furthermore, as the low-intensity intervention was designed with consideration given to limited health service resources, assessment of the overall cost to deliver the interventions, and the cost-effectiveness of each intervention compared to control and to each other, would provide valuable information to guide dissemination of such implementation strategies, and in the design of future implementation interventions. Future research into the costs associated with each intervention is, therefore, warranted. 

The study findings should be interpreted in light of the following strengths and limitations. Strengths include a methodologically sound design, namely an RCT, the blinding of outcome data collectors, and the application of theory for development of the interventions. The strategic use of a three-arm RCT design, resulting from using a common control group for two interventions, allowed for study findings to be generated more rapidly as multiple interventions were tested, rather than sequential testing of each individual intervention [64], and for the optimising of resources associated with conducting RCTs [65,66,67,68]. As resource constraints are not an uncommon barrier to the conduct of large-scale, methodologically rigorous implementation research, employment of multi-arm RCTs in future research would be beneficial. Limitations of the study include a relatively low sample size resulting in insufficient power to detect any differences between the high- and low-intensity interventions. Future research should ensure multi-arm RCTs are fully powered in order to reap the benefits of conducting research using this design. While the methods for assessing menu compliance have previously been employed [18,20], the reliability and validity of this method has not been determined. Furthermore, the research was undertaken in a single region of NSW in which childcare services have been exposed to obesity prevention initiatives for over 10 years [9,27,48]. As such, the sample may not be representative, and the effects of the interventions may not be generalizable to childcare services nationally. It may be hypothesized that services outside of the region may have had lower menu and food group compliance at baseline, therefore enabling the interventions to produce greater effect sizes. Finally, the secondary outcomes and the comparison of outcomes between the high- and low-intensity interventions were not prospectively registered.

## 5. Conclusions

This study is the first three-arm RCT to measure the effectiveness of high- and low-intensity implementation interventions in improving childcare service compliance with nutrition guidelines for the sector. Findings suggest that the interventions did not result in improvements in overall menu compliance with nutrition guidelines. Furthermore, marginal differences in food group compliance and food group servings were found when comparing a low-intensity intervention to control, and a high-intensity intervention to low-intensity. However, findings also suggest a high-intensity implementation intervention may be effective in supporting childcare service implementation of individual food group recommendations, compared to control. Such findings provide important information for policy and practice decision makers responsible for the implementation and sustainability of obesity prevention initiatives in the childcare setting. Future research is warranted to identify strategies effective in increasing overall nutrition compliance with nutrition guidelines, and determine the cost-effectiveness of implementation strategies.

## Figures and Tables

**Table 1:** Summary of the high- and low-intensity implementation strategies.

Implementation Strategy	Description according to ERIC ^a^ [35]	Application within the Interventions according to Proctor [36]	Low- Intensity [20]	High-Intensity [18]
Provision of staff training [37,38,39,40]	Conduct educational meetings: hold meetings targeted toward different stakeholder groups (e.g., providers, administrators, other organizational stakeholders, and community, patient/consumer, and family stakeholders) to teach them about the innovation.	Actor: implementation support officer. Action: A one-day face-to-face menu-planning workshop was provided to service managers and cooks with the aim to improve their knowledge and skills in the application of nutrition guidelines to food preparation and provision. The workshop incorporated both didactic and interactive components including small group discussions, case studies, problem-solving and idea sharing, facilitator feedback and opportunities to practice planning a menu in accordance with guidelines. Experienced implementation support officers and dietitians facilitated the workshop. Target: service managers and cooks. Temporality: one-day face-to-face training workshop at intervention commencement (one week). Dose: One-off workshop.	✓	✓
Provision of resources [25]	Distribute educational materials: distribute educational materials (including guidelines, manuals, and toolkits) in person, by mail, and/or electronically.	Actor: implementation support officer. Action: all intervention services received a resource pack which included the Caring for Children resource [25], menu planning checklists, recipe ideas, budgeting fact sheets, and goal setting and action planning templates, to support guideline implementation. Target: service managers and cooks. Temporality: provided during the one-day face-to-face training workshop at intervention commencement (one week). Dose: one-off provision of resource pack, which could be accessed on an ongoing basis over the six month intervention period.	✓	✓
Audit and feedback [41]	Audit and provide feedback: collect and summarize clinical performance data over a specified time period and give it to providers to monitor, evaluate, and modify provider behaviour.	Actor: trained dietitian. Action: a trained dietitian completed an audit of services two-week menu, with written feedback via email (high-intensity and low-intensity) and verbal feedback via face-to-face support visits (high-intensity only) provided to service managers and cooks. Feedback included overall menu and individual food group compliance with nutrition guidelines, servings of each food group per child per day, and tips for increasing menu compliance. Target: service managers and cooks. Temporality: high-intensity—immediately post-baseline data collection and at three months; low-intensity—immediately post-baseline data collection. Dose: high intensity—twice within the first three months of the intervention period; low intensity—once within the first three months of the intervention period.	✓ (once, written only)	✓ (twice, written and verbal)
Implementation support [42,43]	Provide ongoing consultation: provide ongoing consultation with one or more experts in the strategies used to support implementing the innovation.	Actor: implementation support officer. Action: services were each allocated an implementation support officer to provide tailored and expert advice and assistance to facilitate guideline implementation. Each implementation support officer offered two face-to-face support visits with the service manager and cook, at the service, following the menu planning workshop. In addition, two newsletters were distributed to services. Target: service managers and cooks. Temporality: face-to-face contacts made at two-four weeks following workshop and at three months; newsletters distributed at three months and five months. Dose: twice during the six month intervention period.	X	✓
Securing executive support [44]	Obtain formal commitments: obtain written commitments from key partners that state what they will do to implement the innovation. Mandate change: have leadership declare the priority of the innovation and their determination to have it implemented.	Actor: implementation support officer, service manager. Action: a memorandum of understanding, detailing each party’s responsibilities to implement the nutrition guidelines and participate in the intervention was signed by the implementation support officer, the service manager and the service cook. Service managers were encouraged to communicate support and endorsement of nutrition guideline adherence to other service staff and to update the service nutrition policy accordingly (if required). Targets: service managers, cooks, service staff. Temporality: memorandum of understanding signed within the face-to-face meeting at two–four weeks post workshop. Dose: one-off memorandum of understanding during the first face-to-face contact; ongoing communication of support and endorsement of the guidelines throughout the six months of the intervention period.	X	✓

^a^ ERIC: Expert Recommendations for Implementing Change.

**Table 2:** Baseline characteristics of participating childcare services and service cooks.

Characteristic	Low Intensity (*N* = 24) *n* (%)	High Intensity (*N* = 25) *n* (%)	Control (*N* = 20) *n* (%)
Service operational characteristics			
Average no. of children the service provides food for each day (mean (SD))	54.9 (17.7)	62.4 (23.1)	53.6 (19.9)
Services in high socioeconomic area	7 (29.2)	10 (40.0)	4 (20.0)
Service location			
Major city + inner regional	20 (83.3)	23 (92.0)	17(85.0)
Outer regional/remote Australia	4 (16.6)	2 (8.0)	2 (10.0)
Service cook characteristics			
University or Technical and Further Education (TAFE) qualification	13 (54.2)	13 (52.0)	18 (90.0) *
<40 years of age	9 (42.9) ^a^	7 (29.2) ^b^	5 (26.3) ^c^
>5 years employed within the childcare setting	10 (43.5) ^d^	9 (37.5) ^b^	7 (35.0)
Works ≤20 h per week	4 (17.4) ^d^	2 (8.0)	5 (25.0)

* = *p* < 0.05; ^a^ *n* = 21; ^b^ *n* = 24; ^c^ *n* = 19; ^d^ *n* = 23.

**Table 3:** Overall and individual Australian Guide to Healthy Eating (AGHE) food group menu compliance at baseline and 12-month follow-up ^a.^

Outcome	Baseline			12-Month Follow-Up			Group Interaction Analysis	Low-Intensity vs. Control Pairwise Analysis		High-intensity vs. Control Pairwise Analysis		Low-intensity vs. High-Intensity Pairwise Analysis	
Compliance	Low Intensity (*N* = 24) *n* (%)	High Intensity (*N* = 25) *n* (%)	Control (*N* = 20) *n* (%)	Low Intensity (*N* = 21) *n* (%)	High Intensity (*N* = 19) *n* (%)	Control (*N* = 19) *n* (%)	*p*-Value	Odds Ratio (95% Confidence Interval (CI))	*p*-Value	Odds Ratio (95%CI)	*p*-Value	Odds Ratio (95%CI)	*p*-Value
Overall compliance (5/5 food groups)	0 (0)	0 (0)	0 (0)	0 (0)	3 (15.8)	0 (0)	-	-	-	-	-	^-^	^-^
Compliance with individual food groups													
Vegetables	0 (0)	0 (0)	0 (0)	3 (14.3)	6 (31.6)	0 (0)	0.02	3.77 (0.55;∞)	0.27	10.74 [1.87;∞]	0.02	2.70 [0.47;19.82]	0.35
Fruit	1 (4.2)	4 (16.0)	5 (25.0)	6 (28.6)	10 (52.6)	1 (5.3)	<0.01	7.74 [0.76;408.41]	0.10	18.95 [2.13;944.77]	<0.01	2.44 [0.56;11.37]	0.29
Breads and Cereals	4 (16.7)	3 (12.0)	2 (10.0)	5 (23.8)	5 (26.3)	0 (0)	<0.05	7.42 [1.26;∞]	0.06	8.42 [1.42;∞]	0.04	1.15 [0.22;6.10]	1.00
Meat/meat alternatives	0 (0)	1 (4.0)	0 (0)	4 (19.1)	4 (21.1)	1 (5.3)	0.32	4.10 [0.36;219.93]	0.41	4.93 [0.43;267.03]	0.31	1.21 [0.19;7.79]	1.00
Dairy	9 (37.5)	10 (40.0)	5 (25.0)	9 (42.9)	13 (68.4)	1 (5.3)	<0.01	12.20 [1.37;601.68]	0.02	31.49 [3.47;1596.22]	<0.01	2.67 [0.62;12.64]	0.23
Discretionary	3 (12.5)	0 (0)	0 (0)	7 (33.3)	11 (57.9)	2 (10.5)	<0.01	2.37 [0.29;29.90]	0.61	10.86 [1.77;123.66]	<0.01	4.60 [0.95;26.95]	0.06

^a^ Complete case analysis under intention to treat framework—analysis using all available data for baseline and follow-ups in the group to which they were originally assigned.

**Table 4:** Mean number of food groups compliant with nutrition guidelines, and servings of individual food groups at baseline and 12-month follow-up ^a.^

Outcome	Baseline			12-Month Follow-Up			Group Interaction Analysis	Low-Intensity vs. Control Pairwise Analysis		High-Intensity vs. Control Pairwise Analysis		Low-Intensity vs. High-Intensity Pairwise Analysis	
Measure	Low Intensity (*N* = 24) Mean (SD)	High Intensity (*N* = 25) Mean (SD)	Control (*N* = 20) Mean (SD)	Low Intensity (*N* = 21) Mean (SD)	High Intensity (*N* = 19) Mean (SD)	Control (*N* = 19) Mean (SD)	*p*-Value	Mean Difference (95%CI)	*p*-Value	Mean Difference (95%CI)	*p*-Value	Mean Difference (95%CI)	*p*-Value
Number of food groups compliant	0.71 (0.95)	0.72 (0.79)	0.60 (0.88)	1.62 (1.53)	2.58 (1.98)	0.26 (0.56)	<0.01	1.35 [0.40;2.30]	<0.01	2.29 [1.32;3.26]	<0.01	0.94 [−0.01;21.89]	0.05
Servings of individual food groups													
Vegetables	1.48 (0.54)	1.18 (0.50)	1.05 (0.57)	1.77 (0.67)	2.36 (0.92)	1.32 (0.64)	<0.01	0.36 [−0.14;0.88]	0.17	0.98 [0.48;1.48]	<0.01	0.62 [0.14;1.10]	0.01
Fruit	0.77 (0.19)	0.83 (0.51)	0.91 (0.45)	1.06 (0.36)	1.18 (0.33)	0.86 (0.28)	<0.01	0.22 [0.01;0.43]	0.04	0.34 [0.13;0.55]	<0.01	0.11 [−0.09;0.32]	0.27
Breads and Cereals	2.17 (0.54)	2.00 (0.65)	2.13 (0.72)	2.37 (0.75)	2.34 (0.46)	2.20 (0.74)	0.64	0.17 [−0.24;0.58]	0.41	0.17 [−0.25;0.59]	0.42	0.00 [−0.41;0.41]	0.99
Meat/meat alternatives	0.54 (0.12)	0.55 (0.23)	0.50 (0.18)	0.70 (0.19)	0.78 (0.18)	0.63 (0.23)	0.21	0.05 [−0.07;0.18]	0.39	0.12 [−0.01;0.25]	0.08	0.06 [−0.06;0.19]	0.32
Dairy	1.21 (0.36)	1.19 (0.43)	1.13 (0.54)	1.36 (0.39)	1.39 (0.29)	0.92 (0.32)	<0.01	0.43 [0.21;0.65]	<0.01	0.47 [0.24;0.69]	<0.01	0.04 [−0.18;0.25]	0.75
Discretionary	0.60 (0.45)	0.63 (0.44)	0.65 (0.35)	0.25 (0.31)	0.13 (0.21)	0.68 (0.42)	<0.01	−0.41 [−0.60;−0.22]	<0.01	−0.50 [−0.69;−0.31]	<0.01	−0.09 [−0.28;0.10]	0.34

^a^ Complete case analysis under intention to treat framework—analysis using all available data for baseline and follow-ups in the group to which they were originally assigned.
